# Pan-Genomic Dissection of GH1 β-Glucosidases in *Brassica rapa* Identifies *BrBGLU10* as an Important Regulator of Pollen Development

**DOI:** 10.3390/plants15172579

**Published:** 2026-08-24

**Authors:** Ying Huang, Shanxin Zhong, Tianci Hu, Xingbo Chen, Meng Jiang, Xiangshu Dong

**Affiliations:** School of Agriculture, Yunnan University, Kunming 650091, China; 12024128010@stu.ynu.edu.cn (Y.H.); 12024228026@stu.ynu.edu.cn (S.Z.); hutianci@stu.ynu.edu.cn (T.H.); chenxingbo@mail.ynu.edu.cn (X.C.); jiangmeng_xo9c@stu.ynu.edu.cn (M.J.)

**Keywords:** *Brassica rapa*, β-glucosidase, *BrBGLU10*, pollen development

## Abstract

Glycoside hydrolase family 1 (GH1) β-glucosidases (BGLUs) play diverse roles in plant development and stress responses. However, a comprehensive pan-genomic characterization of this gene family across diverse *Brassica rapa* accessions is still lacking. Here, we conducted a pan-genome-wide analysis of *BGLU* genes across 21 *B. rapa* accessions. A total of 1840 *BGLU* genes were identified and clustered into 57 orthologous gene groups (OGGs), comprising 22 core, 19 dispensable, and 16 private groups. Phylogenetic reconstruction assigned these OGGs to five subgroups, and duplication analysis revealed whole-genome duplication as the predominant driver of family expansion, accounting for 47.51% of duplicated genes. Expression profiling identified two core genes, *BrBGLU10* and *BrBGLU56*, as specifically expressed in fertile floral buds and differentially regulated between fertile and sterile lines. CRISPR/Cas9-mediated knockout of *BrBGLU10* resulted in approximately 36% pollen abortion and drastically reduced seed set upon self-pollination, supporting its important role in pollen development. Collectively, these findings establish *BrBGLU10* as an important regulator of pollen development and a potential target for fertility-related applications via gene editing in *B. rapa* and related *Brassica* crops.

## 1. Introduction

*Brassica rapa* is an economically important crop widely cultivated across East Asia, encompassing diverse morphotypes that include both oilseed and vegetable types [1]. As a typical cross-pollinated *Brassica* species, *B. rapa* exhibits pronounced heterosis, making it an attractive system for hybrid breeding. However, the small size of its floral organs renders artificial pollination for hybrid seed production highly labor-intensive and inefficient. The utilization of male-sterile lines has therefore emerged as a critical strategy to overcome this bottleneck in commercial hybrid seed production. Considerable progress has been made in elucidating the genetic basis of male sterility in *B. rapa*. Several genes governing pollen development and male fertility have been cloned and functionally characterized, including *BrACOS5* (*Brassica rapa acyl-CoA synthetase 5*) [2,3], *Bra2Ms* [4], *Brmmd1* [5], and others. Despite these advances, the molecular mechanisms underlying male sterility in *B. rapa* remain far from fully understood [6]. The genetic and regulatory networks orchestrating pollen development involve numerous genes acting in a spatiotemporally coordinated manner, and many key components, particularly those unique to *Brassica* species or exhibiting functional divergence from their *Arabidopsis thaliana* orthologs, have yet to be identified. Consequently, continued discovery and functional characterization of novel genes involved in pollen development and male sterility is of paramount importance for expanding the repertoire of male-sterility resources and fully harnessing heterosis in *B. rapa* breeding programs.

Glycoside hydrolases (GHs; EC 3.2.1.-) constitute a large and diverse group of enzymes that catalyze the hydrolysis of glycosidic bonds between carbohydrates or between a carbohydrate and a non-carbohydrate moiety [7]. As a central resource for carbohydrate-active enzymes, the CAZy database currently classifies 195 GH families, with GH1 representing one of the most extensively studied and functionally diversified families in plants [8]. Plant GH1 β-glucosidases (BGLUs) participate in a broad spectrum of biological processes, including cell wall modification, phytohormone activation and deactivation, chemical defense against herbivores and pathogens, secondary metabolism, and abiotic stress responses [9,10].

In *A. thaliana*, the GH1 family comprises 48 *BGLU* genes that have been phylogenetically classified into approximately ten subfamilies [11]. Functional characterization reveals that *AtBGLU18* and *AtBGLU33* modulate abscisic acid (ABA) signaling by hydrolyzing ABA-glucose ester (ABA-GE) to release active ABA [12,13], while *AtBGLU25* controls flowering time under low-phosphate conditions via subcellular reprogramming [14]. A substantial subset of GH1 enzymes is dedicated to secondary-metabolite turnover. *AtBGLU1*–*6* and *AtBGLU15* govern flavonol accumulation and flavonol-bisglycoside catabolism [15,16,17,18], *AtBGLU7*–*11* act as acyl-glucose-dependent glucosyltransferases in anthocyanin biosynthesis [19], *AtBGLU21*–*23* hydrolyze scopolin [20], and *AtBGLU12*–*17* are implicated in flavonoid utilization [18]. The myrosinase branch includes *AtBGLU28*/*30*, which mobilize glucosinolates for sulfur reallocation under sulfur deficiency [21,22], and *AtBGLU37*/*38*, whose loss impairs allyl isothiocyanate-mediated stomatal closure [23]; *AtBGLU36* and *AtBGLU39* are myrosinase pseudogenes with stamen- and petal-biased expression [24,25]. *AtBGLU26* (*PEN2*, *Penetration 2*) mediates pre-invasion resistance to *Magnaporthe oryzae* [26], whereas *AtBGLU42* underlies MYB72-dependent coumarin exudation and iron-deficiency responses [27]. Tissue-specific roles are also well documented. *AtBGLU20* is tapetum-enriched and functions downstream of MS1 (Male Sterility 1) in pollen development [28,29]; *AtBGLU34*/*35* are root-tip specific and promote auxin biosynthesis [30]; *AtBGLU45*/*46* participate in coniferin metabolism in stems [31]; and *AtBGLU48* (*SFR2*, *Sensitive to freezing 2*) is required for freezing tolerance [32]. Salt-stress assays further showed that knockout of *AtBGLU1* or *AtBGLU19* decreases NaCl sensitivity, implicating GH1 members in ionic-stress acclimation [33]. Transcriptomic profiling of pollen germination additionally detected dynamic expression of *AtBGLU11*, *12*, *14*, *15*, *16*, *22*, *36*, and *40* [34], underscoring that GH1 diversity extends deeply into reproductive development.

Given the pivotal roles of *BGLUs* in plant secondary metabolism, development, and environmental adaptation, members of this family have been systematically characterized in numerous plant species, including *Medicago truncatula* (51 members) [35], *Pyrus bretschneideri* (50) [36], *Gastrodia elata* (11) [37], and *Brassica napus* (149) [38]. Nevertheless, growing evidence indicates that a single reference genome is insufficient to capture the full extent of intraspecific genetic diversity [39]. In response, the pan-genome concept, originally developed for bacteria, has been increasingly adopted in plant research to systematically explore within-species variation [40,41]. The recent assembly and annotation of multiple high-quality genomes have led to the establishment of a pan-genome database for *B. rapa*, providing a robust foundation for comprehensive gene family analyses and functional investigations [42,43,44].

Previous work, based solely on the Chiifu reference genome (v3.0), identified 64 *BrBGLU* genes in *B. rapa* and suggested that several members might participate in pollen development [28]. However, this single-genome approach could not reveal the presence/absence variations or functional diversification of *BGLU* genes across different *B. rapa* varieties. As a polyploid crop that has undergone lineage-specific whole-genome triplication, *B. rapa* likely harbors considerable genetic variability within its gene families, which calls for a pan-genomic perspective to fully understand the evolutionary dynamics and functional divergence of the *BGLU* family.

In this study, we performed a comprehensive pan-genomic identification and analysis of *BGLU* genes using whole-genome data from 21 diverse *B. rapa* accessions. We systematically investigated the genetic diversity and phylogenetic relationships of *BrBGLU* members across these accessions, and examined their expression profiles in various organs and during pollen development. Furthermore, we functionally characterized *BrBGLU10*, an important candidate gene implicated in pollen development, using CRISPR/Cas9-mediated gene editing. Collectively, our findings address a critical gap in understanding the BGLU family in a polyploid *Brassica* crop and establish *BrBGLU10* as a promising target for male sterility-based applications in hybrid breeding.

## 2. Results

### 2.1. Identification of the BGLUs in the B. rapa

Using HMMER, a total of 1840 *BGLU* genes were identified across 21 *B. rapa* accessions, with an average of approximately 87 genes per accession (Table 1 and Table S1). Among these accessions, MIZ contained the highest number of *BrBGLU* genes (110), while TUA contained the fewest (70). In the reference genome Chiifu (v4.0), 83 *BrBGLU* genes were identified and named in ascending order according to their genomic locations. The BrBGLU protein sequences ranged in length from 59 amino acids (aa) in ECD04 to 2642 aa in BraZ1, with a mean length of 492 aa. Correspondingly, the molecular weight (MW) ranged from 7.41 to 306.12 kDa (mean 56.06 kDa), and the predicted isoelectric point (pI) ranged from 4.38 (OIA) to 9.99 (ECD04), with a mean of 6.74 (Table 1 and Table S1). The *BrBGLU* genes from other accessions were named and classified based on their BLASTp hits to the reference BrBGLUs in Chiifu (v4.0) (Table S1). Domain architecture analysis revealed that, among the 289 incomplete BrBGLUs, 170 lacked the N-terminal region and 89 were truncated at the C-terminus. Notably, 30 members retained the complete PF00232 domain despite terminal deletions, suggesting that they may still encode functional enzymes with altered subcellular targeting or regulatory properties (Table S1).

**Figure 1:**
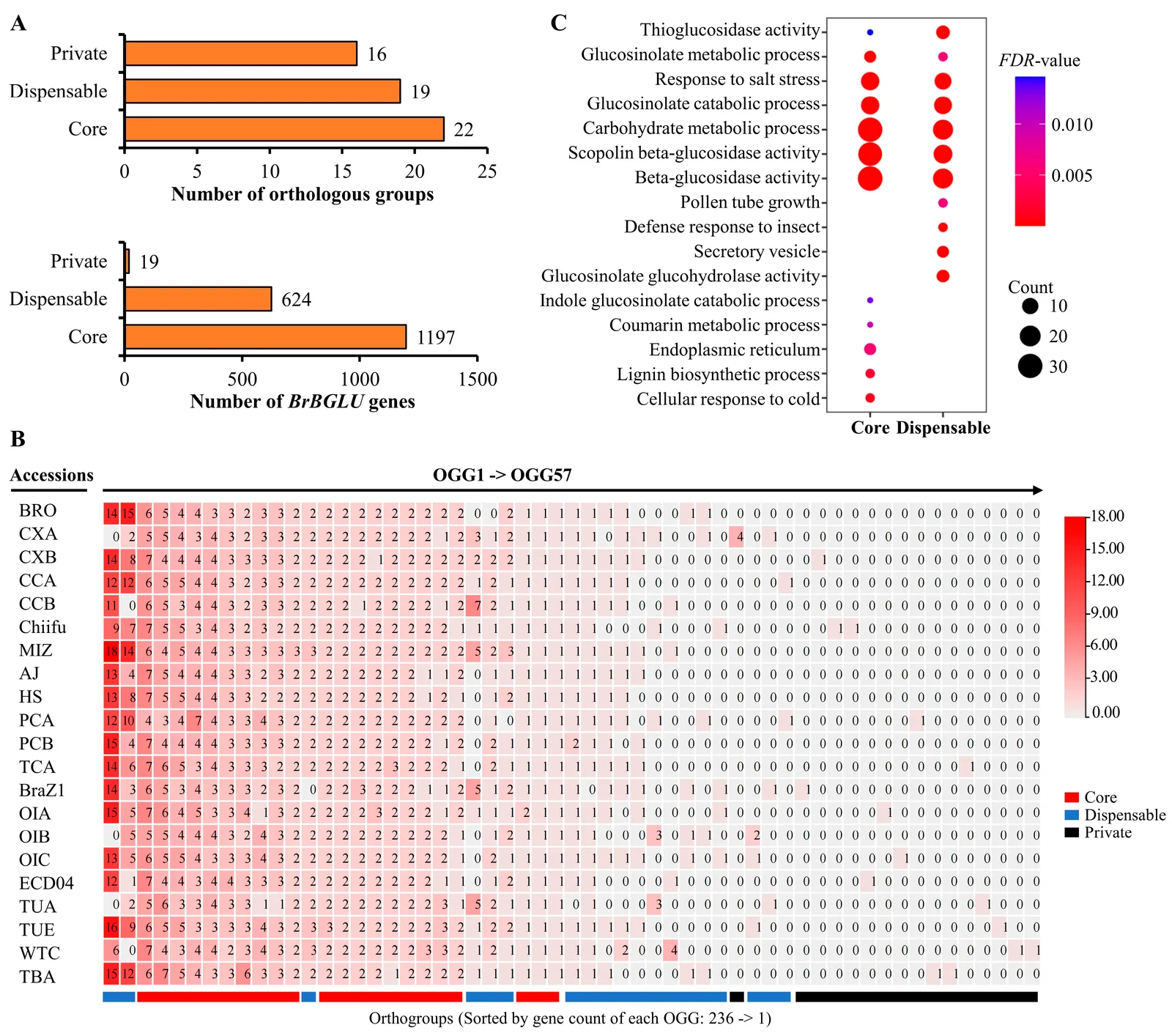
Characterization and classification of *BrBGLU* genes into core, dispensable, or private categories across 21 *Brassica rapa* accessions. (**A**) Composition of orthologous gene groups (OGGs). The upper and lower bar plots represent the number of OGGs and *BrBGLU* genes, respectively, classified into core, dispensable, and private categories. (**B**) Occurrence of BrBGLU orthologous gene groups (OGGs) across *B. rapa* accessions. A total of 57 OGGs, numbered sequentially as OGG1 to OGG57, are shown. The heatmap illustrates the distribution of each OGG across the 21 accessions, where filled cells indicate presence and empty cells indicate absence. The numerical value within each cell denotes the gene count for that OGG in the corresponding accession. The color scale (from light to dark) reflects the gene copy number. The red lines indicated the core OGGs, the blue lines indicated the dispensable OGGs, the black lines indicated the private OGGs. (**C**) Gene Ontology (GO) enrichment analysis of core, dispensable, and private *BrBGLUs*.

A total of 57 orthologous gene groups (OGGs) were identified and classified into three categories based on their distribution frequency across the 21 genomes: core OGGs (present in all genomes, *n* = 22), dispensable OGGs (present in 2 to 20 genomes, *n* = 19), and private OGGs (unique to a single genome, *n* = 16) (Figure 1A; Table S2). Among these categories, core OGGs contained the largest number of genes, with 1197 genes in total, accounting for 65.05% of the entire gene family, whereas private OGGs contained the fewest, with only 19 genes (Figure 1A). Based on gene content, the 57 *B. rapa* BGLU OGGs were designated OGG1 through OGG57 (Figure 1B; Table S2). Examination of the presence–absence patterns of *BrBGLUs* across accessions revealed that gene copy number within each OGG did not strictly correlate with its pan-genome category. For instance, OGG1 and OGG2, which harbored the highest gene counts, were both classified as dispensable rather than core OGGs (Figure 1B).

GO enrichment analysis revealed that both core and dispensable *BrBGLUs* were significantly enriched in terms associated with glucosinolate metabolic process, response to salt stress, glucosinolate catabolic process, carbohydrate metabolic process, scopolin beta-glucosidase activity, and beta-glucosidase activity (Figure 1C). Further analysis demonstrated that core *BrBGLUs* were additionally enriched in biological processes related to indole glucosinolate catabolic process, coumarin metabolic process, lignin biosynthetic process, and cellular response to cold (Figure 1C). In contrast, dispensable *BrBGLUs* were specifically enriched in pollen tube growth and defense response to insect (Figure 1C). These findings suggest that core and dispensable *BrBGLUs* share conserved functions in primary and secondary metabolic processes, whereas their roles in abiotic stress responses and developmental processes are distinct.

### 2.2. Phylogenetic Classification of the BrBGLUs in B. rapa

**Figure 2:**
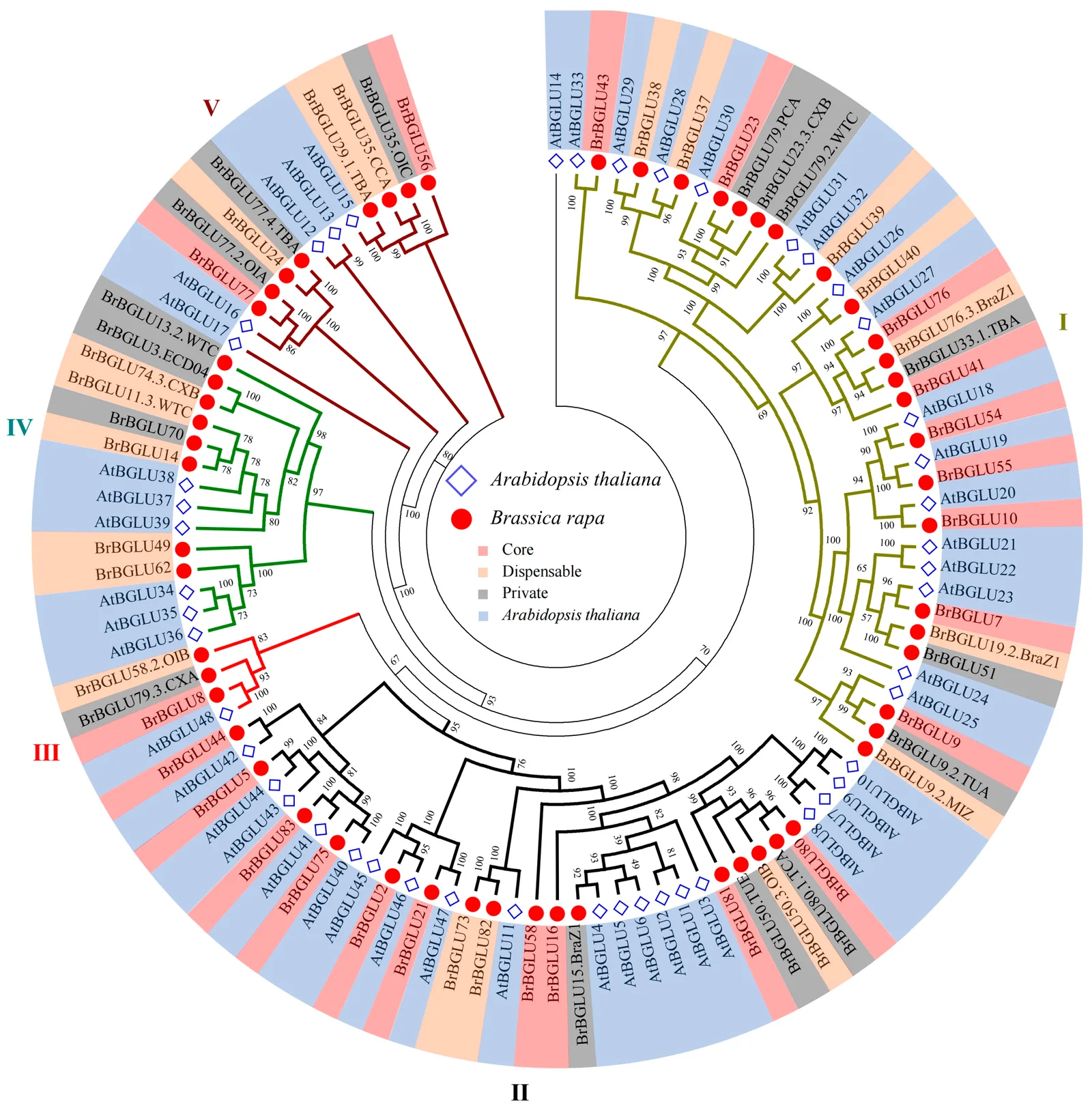
Phylogenetic classification of *B. rapa* and *Arabidopsis* BGLUs. A maximum-likelihood phylogenetic tree was constructed based on full-length amino acid sequences of BGLU proteins. Representative sequences from 57 *B. rapa* orthologous gene groups (OGGs) were included, with *Arabidopsis* BGLUs (highlighted in blue) serving as reference for subfamily classification. BrBGLU OGGs were categorized into three classes according to their presence across 21 *B. rapa* genomes: core (present in all accessions, red), dispensable (present in a subset of accessions, orange), and private (unique to a single accession, gray). Distinct subgroups are indicated by different branch colors and labeled accordingly. I–V in the figure indicate the classification of all BGLU proteins into five subgroups.

To investigate the evolutionary status of BrBGLUs in *B. rapa*, with particular emphasis on dispensable BrBGLUs and those absent from the reference genome Chiifu (v4.0), a phylogenetic analysis was conducted on the 57 BrBGLU OGGs. For each OGG containing a BGLU in Chiifu, the Chiifu sequence was used as the representative. For the 23 OGGs absent in Chiifu, the longest BrBGLU sequence from each respective OGG across the remaining 20 *B. rapa* genomes was selected. *Arabidopsis* BGLUs (AtBGLUs) were employed as references for subfamily classification. The 57 *B. rapa* pangenes were classified into five subgroups (Figure 2). Previously, AtBGLUs and BrBGLUs were classified into ten subgroups [28]. In the current study, subgroups GH1-f, GH1-g, GH1-h, and GH1-I were consolidated into subgroup I, while subgroups GH1-a, GH1-b, and GH1-c were merged into subgroup II. Subgroups GH1-j, GH1-e, and GH1-d corresponded to subgroups III, V, and IV, respectively (Figure 2). In addition, the AtBGLU14 which belong to the subgroup GH1-e were not classified into any subgroup in current study.

### 2.3. Analysis of Duplication and Expansion Patterns in BrBGLU Genes

**Figure 3:**
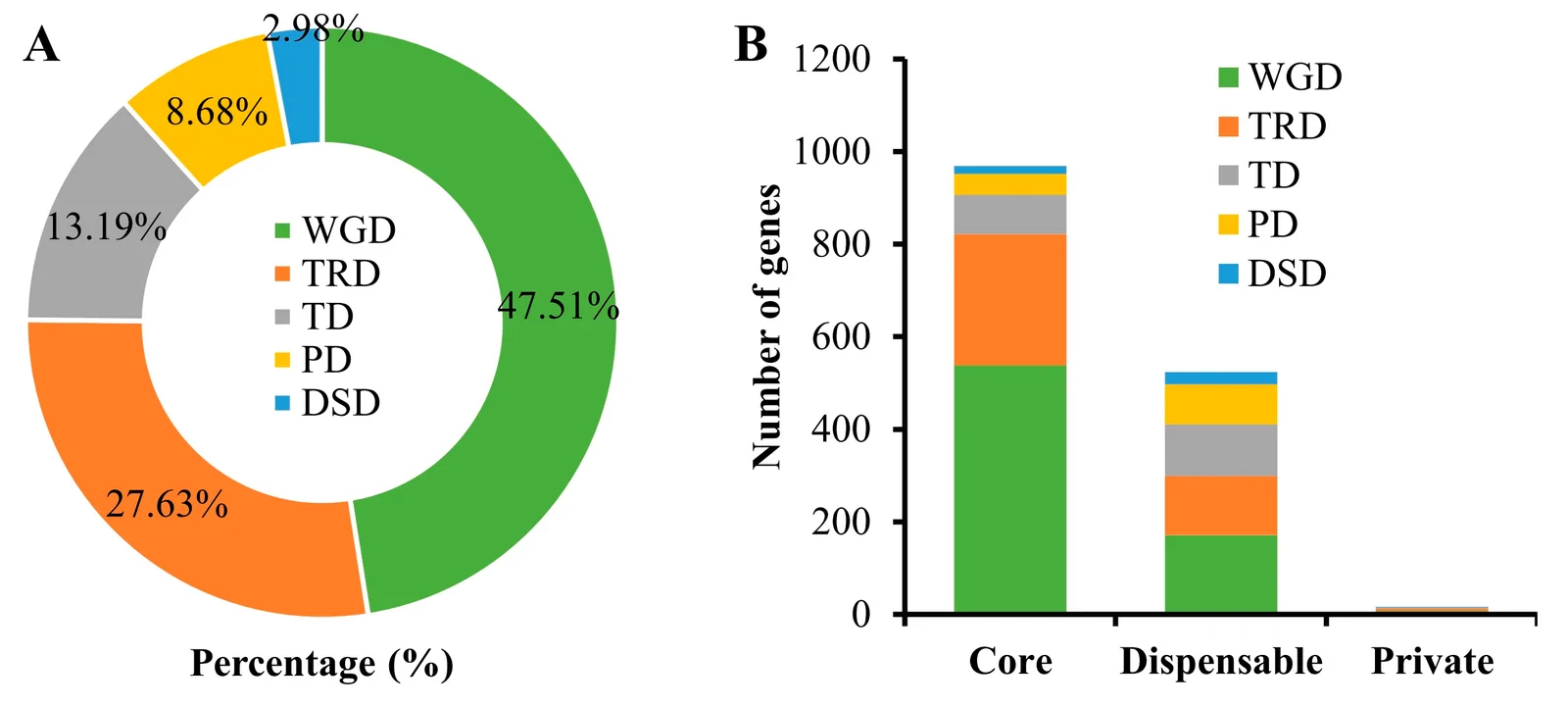
Duplication analysis of the *BrBGLU* gene family in *B. rapa*. (**A**) Pie chart showing the distribution of *BrBGLU* gene duplication types. (**B**) Stacked bar chart displaying *BrBGLU* gene duplications grouped by category. Abbreviations: WGD, whole-genome duplication; TRD, transposed duplication; TD, tandem duplication; PD, proximal duplication; DSD, dispersed duplication.

To elucidate the expansion mechanisms of *BGLUs* in *B. rapa*, the gene duplication patterns across 21 accessions were analyzed. A total of 1509 duplicated *BrBGLU* genes were identified and classified into five categories. Among these, whole-genome duplication (WGD) accounted for the largest proportion, with 717 genes (47.51%), followed by transposed duplication (TRD) with 417 genes (27.63%). Tandem duplication (TD), proximal duplication (PD), and dispersed duplication (DSD) comprised 199 (13.19%), 131 (8.68%), and 45 (2.98%) genes, respectively (Figure 3A; Table S3).

Examination of duplication patterns across the three *BrBGLU* categories (core, dispensable, and private) revealed broadly similar distributions (Figure 3B). In the core gene set, WGD-derived duplications were the most prevalent, followed by TRD and TD. The dispensable category was also predominantly characterized by WGD, with TRD and TD ranking second and third, respectively. Similarly, in the private category, WGD again constituted the largest group.

### 2.4. Tissue Expression of BrBGLUs Reveals Their Potential Functions During Pollen Development

**Figure 4:**
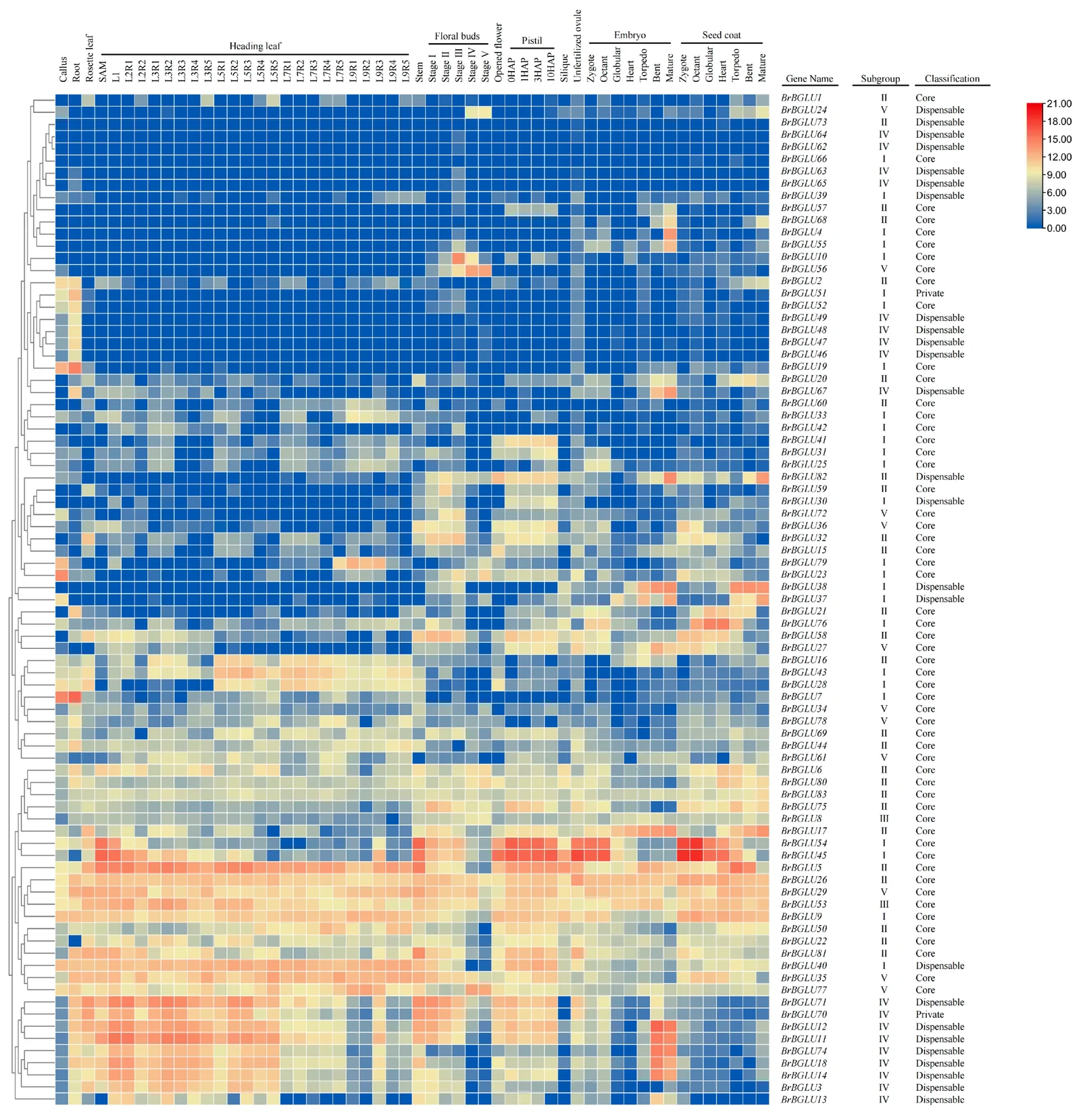
Expression of *BrBGLUs* in different tissues and organs of *Brassica rapa*. Expression data were subjected to log_2_(TPM + 1) normalization. Comparisons of stem leaf, stem, and root tissues were conducted by comparison against seven-week-old Chiifu (*B. rapa* cv. Chiifu) plants. Flower tissue was also generated from blooming plants without floral shoots, while silique tissues were obtained 15 days after pollination. Further, 24 samples from the heading leaves of eleven-week-old plants were collected. All leaves from the heading leaf samples were divided into eleven whorls extending from the inside to the outside. Leaves with length < 2 cm and shoot apical meristem (SAM) are identified as SAMs. Leaves from whorls one, two, three, five, seven, and nine were identified as L1, L2, L3, L5, L7, and L9, respectively. L2 samples were divided into leaf petiole (L2R2) and leaf blade (L2R1) samples, while L3, L5, L7, and L9 were divided into regions including the top region (R1), outer margin region (R2), middle region of the blade (R3), top region of the petiole (R4), and middle region of the petiole (R5). Stage I to V indicate the floral buds representing the pollen mother cells, tetrad, uninucleate pollen, binucleate pollen, and mature pollen stages, respectively. 1 HAP, 3 HAP, and 10 HAP indicate pistils at 1, 3, and 10 h after pollination in the fertile line, respectively.

To survey the transcriptional repertoire of *BrBGLU* family members, RNA-seq data were obtained from 54 samples spanning 12 tissue categories, including callus, root, stem, stem leaf, flower, silique, head leaf (*n* = 24), floral buds at five progressive stages, pistils (*n* = 4), unfertilized ovules, embryos (*n* = 7), and seed coats (*n* = 7), and were aligned to the reference genome Chiifu (v4.0) (Figure 4). Profiling of transcript abundance revealed marked tissue-preferential expression among *BrBGLUs*. A set of seven genes, *BrBGLU52*, *BrBGLU51*, *BrBGLU49*, *BrBGLU48*, *BrBGLU47*, *BrBGLU46*, and *BrBGLU19*, exhibited preferential accumulation in callus and root tissues, whereas *BrBGLU4*, *BrBGLU55*, *BrBGLU57*, and *BrBGLU68* were predominantly enriched in mature embryos. Additionally, two core genes, *BrBGLU10* (stages II–IV) and *BrBGLU56* (stages II–V), displayed stage-specific expression fluctuations during floral bud development, hinting at their involvement in reproductive processes (Figure 4). To further investigate the functional relevance of *BrBGLU10* and *BrBGLU56*, RNA-seq datasets from five independent genic male-sterile lines were re-analyzed. Among the 83 *BrBGLU* genes, 43 showed differential expression between fertile and sterile floral buds in at least one male-sterile line. Specifically, 17, 18, 8, 15, and 35 *BrBGLU* genes were differentially expressed in the ms77B, FT, AB, AB01, and Bcajh97-01A/B lines, respectively (Table S4; Figure S1). *BrBGLU10* was consistently up-regulated in fertile floral buds across all five lines, whereas *BrBGLU56* showed up-regulation in fertile buds of the ms77B, FT, AB01, and Bcajh97-01A/B lines, but not in AB. In addition to these two target genes, several *BrBGLU* genes, including *BrBGLU6*, *BrBGLU23*, *BrBGLU24*, *BrBGLU37*, *BrBGLU80*, and *BrBGLU83*, exhibited differential expression across multiple male-sterile lines (Figure S1).

To explore potential co-expression relationships between the two target genes and other differentially expressed *BrBGLUs*, Pearson correlation coefficients (PCCs) were calculated based on TPM values across the five male-sterile lines. *BrBGLU80* showed high co-expression with *BrBGLU10* (PCC = 0.90), while *BrBGLU83* was highly co-expressed with *BrBGLU56* (PCC = 0.91) (Table S4). These results suggest that functional redundancy among *BrBGLU* members may exist during pollen development. Therefore, given that the primary difference between fertile and sterile floral buds in these lines is the presence of pollen grains [45,46,47,48,49], and considering the floral organ-preferential expression of *BrBGLU10* and *BrBGLU56* and their status as core genes (Figure 4), these two genes represent ideal candidates for further functional characterization.

### 2.5. Verification of the Function of BrBGLU10 During Pollen Development

**Figure 5:**
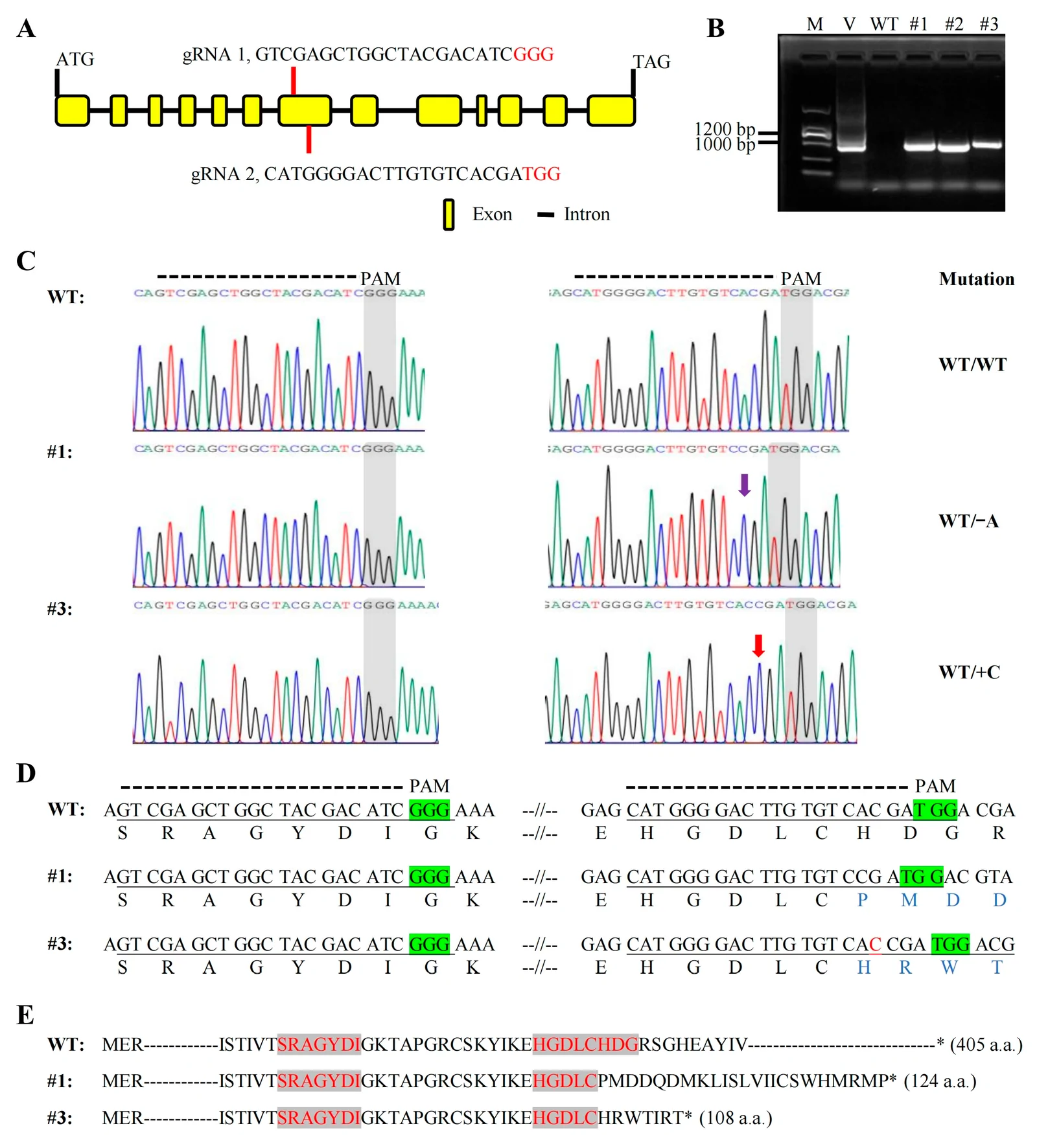
CRISPR/Cas9-mediated mutagenesis of *BrBGLU10*. (**A**) Schematic representation of the *BrBGLU10* genomic architecture, indicating the positions of two designed sgRNA target sequences (gRNA1 and gRNA2) relative to the PAM motifs (red letters). (**B**) PCR-based genotyping of three independent T_0_ transgenic lines (#1–#3), with empty vector control (V) and DNA marker (M) shown for reference. (**C**) Sanger sequencing chromatograms of the gRNA1- and gRNA2-flanking regions from T_1_ plants, with red arrows denoting nucleotide insertions and purple arrows indicating deletions. (**D**) Multiple sequence alignment of the sgRNA-targeted region across different mutant alleles, with the PAM sequence shaded in green. (**E**) Translation of the mutated sequences reveals frameshift-induced premature stop codons in *BrBGLU10*, as shown by deduced amino acid sequences from lines carrying an A deletion and a C insertion; target-site residues are highlighted in grey with red lettering. The asterisk (*) indicates a stop codon.

To investigate the function of *BrBGLU10*, CRISPR/Cas9-mediated gene editing was employed to generate knockout mutants (Figure 5A,B). Approximately 900 cotyledon explants of *B. rapa* GT-24 were infected with *Agrobacterium tumefaciens* harboring a recombinant binary vector. Following selection, four buds were obtained, corresponding to a differentiation rate of 0.44%. A total of four shoots regenerated into rooted plantlets, of which three were successfully transferred to the greenhouse and survived to maturity (designated #1 to #3). The presence of the transgene in T_0_ plants was confirmed by PCR using primers *BrBGLU10cas9*-F and *BrBGLU10cas9*-R, which amplify a T-DNA region containing the *Cas9* expression cassette and the sgRNA sequence (Table S5; Figure 5B). All three plants were confirmed as transgenic, yielding a transformation efficiency of 0.33% relative to the initial number of explants. To verify targeted mutagenesis, the *BrBGLU10* genomic locus was amplified from transgenic plants using primers *BrBGLU10GT*-F and *BrBGLU10GT*-R, and the PCR products were subjected to Sanger sequencing. Sequencing results revealed that all three T_0_ plants carried distinct editing events, with all sequenced clones showing identical mutations, confirming that all three mutants were homozygous. After flowering, bud pollination was performed on T_0_ plants to obtain T_1_ seeds. Five and three T_1_ seeds were obtained from lines #1 and #3, respectively, whereas no seeds were obtained from line #2. Sequencing of the mutated region in T_1_ plants confirmed that the mutations were stably inherited and identical to those observed in the corresponding T_0_ plants, including a single nucleotide deletion (A) in line #1 and a single nucleotide insertion (C) in line #3. These frameshift mutations resulted in premature stop codons at various positions, leading to the production of truncated proteins (Figure 5C–E).

**Figure 6:**
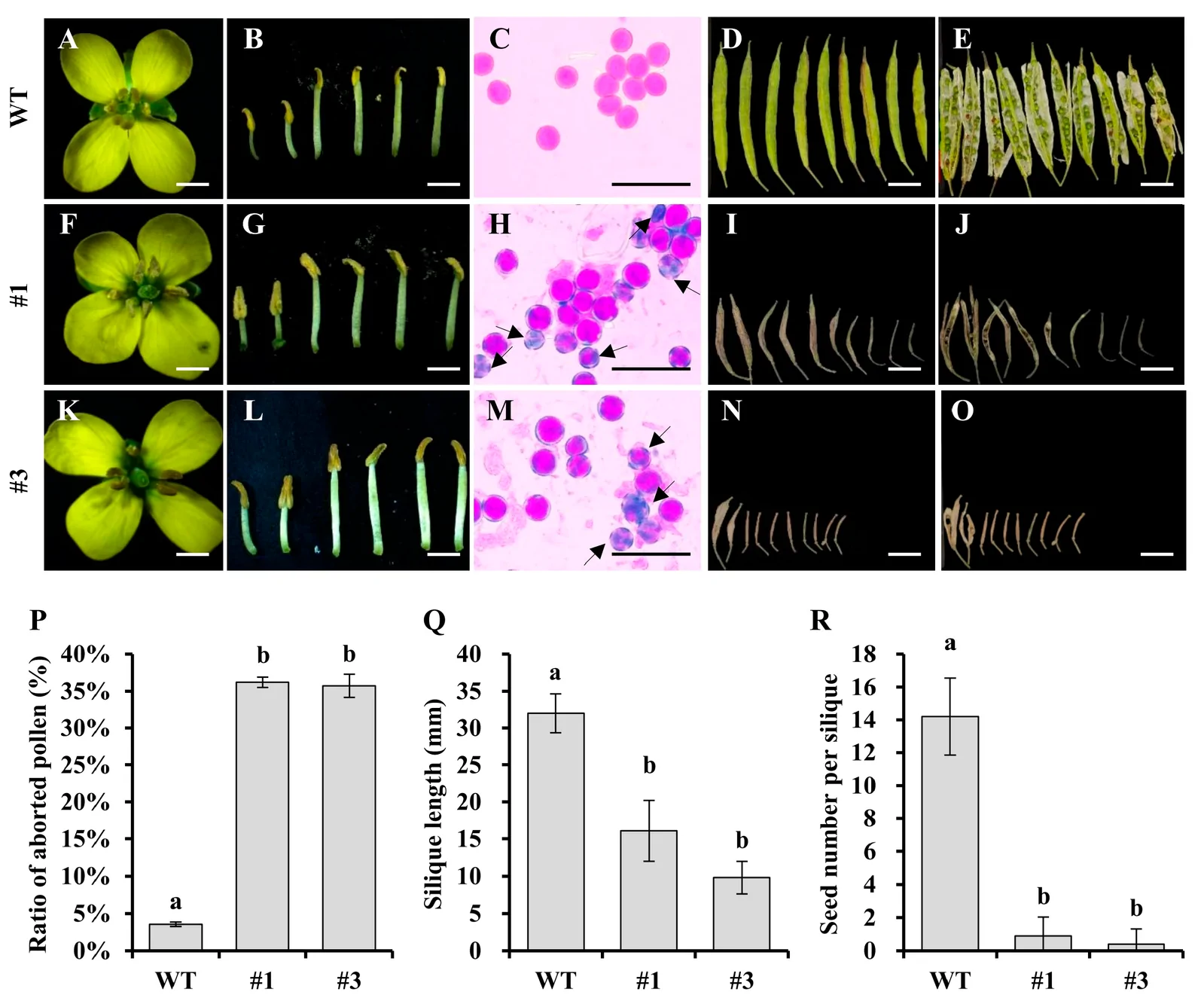
Phenotypic comparison of wild-type (WT) and *BrBGLU10* mutant lines. (**A**–**E**) Morphological characterization of WT plants: (**A**) flower, (**B**) stamen, (**C**) mature pollen grains, (**D**) intact silique, and (**E**) opened silique. (**F**–**J**) Corresponding phenotypes of line #1. (**K**–**O**) Corresponding phenotypes of line #3. Black arrows indicate aborted pollen grains. (**P**) Percentage of aborted pollen grains. (**Q**) Silique length (cm) of WT, line #1, and line #3. (**R**) Seed number per silique in WT, line #1, and line #3. Data are presented as mean ± SD (*n* ≥ 3 biological replicates). Different letters above the bars indicate statistically significant differences among groups (one-way ANOVA, *p* ≤ 0.05). Scale bars: 2 mm (flowers and stamens (**A**,**B**,**F**,**G**,**K**,**L**) and pollen panels (**C**,**H**,**M**)) and 1 cm (siliques (**D**,**E**,**I**,**J**,**N**,**O**)).

The T_1_ *BrBGLU10* mutants exhibited normal vegetative growth but contained approximately 36% aborted pollen grains compared with wild-type plants (Figure 6). Alexander’s staining revealed that the pollen wall of the mutants showed a darker blue coloration relative to the wild type, indicating reduced pollen viability. Furthermore, self-pollination of T_1_ plants resulted in significantly shorter siliques than those of the wild type, and only a few seeds were obtained per silique. Taken together with the expression pattern of *BrBGLU10* and the mutant phenotypes, our results indicate that *BrBGLU10* is important for normal pollen development and male fertility.

## 3. Discussion

### 3.1. Diversity of BrBGLUs in the Pan-Genome of B. rapa

The pan-genome approach captures substantially more genetic diversity than a single reference genome, thereby providing a more comprehensive foundation for gene family characterization and functional investigation [50]. In the present study, we identified *BrBGLU* genes across 21 *B. rapa* accessions (Table S1 and Figure 1). While a previous study based on the Chiifu reference genome (v3.0) reported 64 *BrBGLU* genes [28], our pan-genomic survey expanded this number to 1840 potential *BrBGLU* genes across the 21 accessions (Table 1 and Table S1). Notably, 83 *BrBGLU* genes were identified in the updated Chiifu reference genome (v4.0) (Table 1), which likely reflects improvements in genome assembly quality and annotation completeness. Of the identified BrBGLUs, 289 (15.7%) were incomplete at the N- or C-terminus. Although these models passed multiple validation steps (HMMER, BLASTP, and CDD), their truncated nature warrants caution when interpreting gene copy-number variation and functional predictions, as fragmented assemblies can introduce substantial errors in gene annotation. Future experimental validation, including RT-PCR and full-length cDNA sequencing, will be required to confirm the expression and integrity of these incomplete models.

The copy number of *BrBGLU* genes exhibited substantial variation across accessions, ranging from 70 in TUA to 110 in MIZ (Table 1). Notably, only three of the 21 analyzed genomes, Chiifu (v4.0), AJ, and HS, represent telomere-to-telomere (T2T) assemblies [42,43,44]. Although T2T assemblies typically resolve gene space more completely and often yield higher gene counts than conventional approaches, the three T2T genomes in this study harbored only 82–86 *BrBGLU* genes. These counts were not markedly higher than those derived from many non-T2T assemblies, suggesting that assembly quality alone cannot fully account for the observed copy number variation. Instead, this disparity likely reflects a confluence of factors, including genuine biological divergence among accessions, methodological discrepancies in annotation pipelines, and lineage-specific patterns of gene gain or loss. Among the 21 accessions, we identified 1197 core genes, 624 dispensable genes, and 19 private genes (Table S1 and Figure 1). Accession CXA harbored the highest number of private genes (four), followed by Chiifu, TBA, and WTC (two each), while CXB, OIA, OIC, BraZ1, PCA, TCA, TUA, and TUE each contained one private gene (Table S1). These observations suggest that *BrBGLU* genes have undergone differential evolution across *B. rapa* accessions, potentially reflecting adaptive divergence in response to diverse environmental conditions and selection pressures.

Collectively, our pan-genomic analysis not only substantially expands the known repertoire of *BrBGLU* genes beyond the single-reference framework but also reveals extensive presence–absence variation within this gene family, underscoring the necessity of a pan-genome perspective for understanding the functional diversification of GH1 β-glucosidases in *B. rapa*.

### 3.2. Phylogenetic Analysis of BrBGLUs

The phylogenetic analysis revealed that *BrBGLUs* and *AtBGLUs* collectively clustered into five major subgroups (I–V) (Figure 2), suggesting a shared evolutionary origin between the two species, despite subsequent lineage-specific duplication, gene loss, and functional diversification. Notably, while a previous study classified *AtBGLU* and *BrBGLU* into ten subfamilies based on the Chiifu reference genome (v3.0) [28], the present pan-genomic analysis consolidated several of these subfamilies. Specifically, subgroups GH1-f, GH1-g, GH1-h, and GH1-i were merged into subgroup I; subgroups GH1-a, GH1-b, and GH1-c were merged into subgroup II; and subgroups GH1-j, GH1-e, and GH1-d corresponded to subgroups III, V, and IV, respectively (Figure 2). This reclassification likely reflects both the expanded gene repertoire uncovered by the pan-genomic approach and improved phylogenetic resolution.

Functional inference based on *Arabidopsis* orthologs provided insights into the potential roles of each *BrBGLU* subgroup. Subgroup I appears to be predominantly associated with stress responses and secondary metabolism. For instance, *AtBGLU21*–*23*, which hydrolyze scopoline, a root-specific metabolite involved in defense against pathogens and abiotic stresses, fall within this subgroup [20]. Similarly, *AtBGLU18* and *AtBGLU33*, which hydrolyze glucose-conjugated abscisic acid (ABA-GE) to elevate ABA levels and enhance drought tolerance, are also members of subgroup I [12,13]. *AtBGLU26* (*PEN2*), which mediates pre-invasion resistance to *Magnaporthe oryzae* [26], and *AtBGLU19*, which modulates salt stress tolerance [33], further support the association of this subgroup with environmental stress adaptation.

Subgroup II encompasses genes involved in flavonoid metabolism and structural development. *AtBGLU1*–*6* contribute to flavonol accumulation, *AtBGLU7*–*11* function as anthocyanin glucosyltransferases [15,16,19], and *AtBGLU45*/*46* participate in coniferin metabolism in stems [31]. Subgroup III contains only *AtBGLU48* (also known as *SFR2*), which is required for freezing tolerance [32]. Subgroup IV includes genes associated with auxin biosynthesis (*AtBGLU34* and *AtBGLU35*) and isothiocyanate-mediated stomatal closure (*AtBGLU37* and *AtBGLU38*) [23,30]. Subgroup V comprises genes involved in flavonol bisglycoside metabolism, such as *AtBGLU11*, *AtBGLU15*, and *AtBGLU16* [18]. These functional associations provide a framework for prioritizing *BrBGLU* candidates for future functional studies, particularly those with potential roles in stress tolerance and secondary metabolism.

### 3.3. Duplication Analysis of BrBGLUs

Gene duplication serves as a primary engine driving genomic innovation, with both whole-genome duplication (WGD) and tandem duplication playing important roles in plant genome evolution [51]. Following duplication, the resulting paralogs typically undergo three possible evolutionary trajectories: pseudogenization, neofunctionalization, or subfunctionalization [52]. Analysis of the *BrBGLU* family revealed that whole-genome duplication (WGD) was the dominant mechanism underlying gene family expansion, contributing 717 genes (47.51%) to the total duplicated genes, followed by transposed duplication (TRD) with 417 genes (27.63%) (Figure 3). Tandem duplication (TD), proximal duplication (PD), and dispersed duplication (DSD) comprised smaller fractions, with 199 (13.19%), 131 (8.68%), and 45 (2.98%) genes, respectively. This pattern is consistent with the known evolutionary history of *B. rapa*, which has undergone lineage-specific whole-genome triplication (WGT) [53]. WGD events provide hundreds to thousands of retained duplicates in a single event, serving as a major source of genetic material for gene family expansion and functional diversification. The predominance of WGD-derived *BrBGLU* genes suggests that the ancient polyploidization event has played a substantial role in shaping the current genomic landscape of this gene family in *B. rapa*.

Previous studies have identified ten *BrBGLU* gene clusters in the Chiifu genome (v3.0), suggesting that tandem duplication also contributed to the expansion of the *BrBGLU* family in *B. rapa* [28]. In current study, the relatively lower contribution of tandem duplications to the *BrBGLU* family expansion (13.19%) contrasts with observations in some other plant gene families, where tandem duplication is often the predominant mode of expansion [54]. This discrepancy may reflect lineage-specific evolutionary dynamics following whole-genome triplication (WGT) in *B. rapa* [53]. It has been demonstrated that tandem arrays in *B. rapa* were dramatically fractionated after WGT, when compared either to non-tandem genes within the *B. rapa* genome or to tandem arrays in closely related species that have not experienced recent polyploidization. Thus, the extensive WGT event in *B. rapa* may have led to the preferential loss or masking of tandemly duplicated *BrBGLU* copies, while simultaneously providing a large reservoir of WGD-derived duplicates. Collectively, these observations indicate that the expansion of the *BrBGLU* family has been primarily driven by whole-genome triplication, with subsequent functional divergence of the retained duplicates likely contributing to the metabolic versatility characteristic of *Brassica* species.

### 3.4. Identification of BrBGLUs Related to Pollen Development

A growing body of evidence from *Arabidopsis* has linked multiple GH1 family members to male gametophyte development and pollen germination. Transcriptomic profiling revealed that *AtBGLU11*, *12*, *14*, *15*, *16*, *22*, *36*, and *40* exhibit dynamic expression changes during pollen germination [34], while *AtBGLU36* displays pollen-specific expression [25]. *AtBGLU20*, in particular, has been implicated in pollen wall and exine formation based on its co-expression with genes involved in sporopollenin biosynthesis and tapetal development [28]. Beyond *Arabidopsis*, functional studies in rice and other plant species have also demonstrated the involvement of β-glucosidases in pollen development, suggesting a conserved role for this enzyme family in male reproduction across angiosperms [55,56].

In *B. rapa*, our transcriptomic analysis revealed that approximately half of the *BrBGLU* genes annotated in the Chiifu reference genome (v4.0) are expressed during floral bud development, with *BrBGLU10* and *BrBGLU56* exhibiting pronounced stage-specific and tissue-preferential expression patterns (Figure 4). Furthermore, re-analysis of RNA-seq data from five independent genic male-sterile lines identified 17, 18, 8, 15, and 35 differentially expressed *BrBGLU* genes in the ms77B, FT, AB, AB01, and Bcajh97-01A/B lines, respectively (Table S4; Figure S1). This set is substantially larger than a previous report that identified 12 *BrBGLU* genes with more than twofold expression changes between fertile and sterile floral buds [28], further supporting the view that the *BrBGLU* family represents a valuable genetic resource for engineering male sterility in *B. rapa*.

Among the pollen-associated *BrBGLU* members, *BrBGLU10* emerged as a particularly compelling candidate based on its expression profile and sequence homology. Previous work in *Arabidopsis* established *AtBGLU20* (also known as *ATA27*) as a critical regulator of pollen development. Knockdown of *AtBGLU20* resulted in the production of defective pollen grains, and co-expression analysis further supported its role in this process [28]. *AtBGLU20* was subsequently identified as a target of *MS1*, a key transcription factor that orchestrates the expression of sporophytic pollen coat protein genes downstream of the *DYT1*-*TDF1*-*AMS*-*MS188*-*MS1* regulatory cascade [57]. *MS1* directly regulates the expression of several pollen coat protein genes, including three lipase proteins (*EXL4*, *EXL5*, and *EXL6*) and *ATA27*/*BGLU20* [57]. In the present study, *BrBGLU10* was identified as the putative ortholog of *AtBGLU20* (Figure 2). Both proteins belong to the glycoside hydrolase family 1 (GH1) and contain the conserved glycoside hydrolase family 1 domain. The similarity of their conserved domains is 83.23%, indicating that they share a similar protein structure.

CRISPR/Cas9-mediated knockout of *BrBGLU10* resulted in approximately 36% aborted pollen grains. Although the remaining pollen grains appeared morphologically normal, self-crossing experiments confirmed that they were non-functional, as evidenced by drastically reduced seed set (Figure 5 and Figure 6). Alexander’s staining further revealed that the pollen wall of *BrBGLU10* mutants exhibited enhanced dark blue coloration relative to wild-type controls (Figure 6), a phenotype consistent with the previously documented role of *AtBGLU20* in pollen exine formation and its co-expression with genes governing pollen coat and exine development. Given the high sequence and domain conservation, together with the similar pollen phenotypes observed in *AtBGLU20* knockdown and *BrBGLU10* knockout lines, we infer that both proteins have an important function in pollen wall formation. *AtBGLU20* is one of the highly abundant proteins identified in the *Arabidopsis* pollen coat, and its expression is dependent on *MS1* and *MS188*. By analogy, *BrBGLU10* may play a similar role in pollen wall formation in *B. rapa*, potentially by participating in the hydrolysis of glycosylated substrates required for pollen coat development or exine deposition. However, the precise physiological substrates of *BrBGLU10* remain to be experimentally determined, and the extent to which its function is fully conserved with *AtBGLU20* awaits further investigation through complementary experiments such as biochemical activity assays and subcellular localization verification.

Collectively, these findings establish a potential functional link between *BrBGLU10* and the conserved MS1-mediated regulatory network that governs pollen wall development and male fertility. We propose that *BrBGLU10* represents an important component of the pollen developmental program in *B. rapa*, and its functional characterization not only extends the known roles of GH1 β-glucosidases in plant reproduction but also provides a potential genetic target for fertility-related breeding strategies in *Brassica* crops.

## 4. Material and Methods

### 4.1. Identification of BrBGLUs and Phylogenetic Tree Construction

To identify BrBGLUs across the 21 *B. rapa* accessions, all predicted protein sequences were downloaded from the BRAD database (http://brassicadb.cn/, accessed on 16 August 2025) or BnIR (https://yanglab.hzau.edu.cn/BnIR, accessed on 16 August 2025). The accessions analyzed included Broccolieto (BRO), Caixin (CXA and CXB), Chinese cabbage types (CCA, CCB, and Chiifu), Sarsons (BraZ1, OIA, OIB, and OIC), turnips (TUA, TUE, and ECD04), Pak choi (AJ, HS, PCA, PCB, and TCA), Wutacai (WTC), Japanese morphotype (mizuna), and an unknown type (TBA, originating from Tibet, China) [42,43,44]. To identify candidate BrBGLU proteins, the predicted proteome of each *B. rapa* accession was searched against the Hidden Markov Model (HMM) profile of the Pfam 32.0 database using HMMER (version 3.1b2) [58]. Protein sequences harboring the PF00232 domain with an *E*-value below 1 × 10^−5^ were retained as initial candidates. To further validate their identity, these candidates were used as queries in BLASTP searches against the *Arabidopsis thaliana* proteome, and only those whose best hit corresponded to a known AtBGLU were kept. All candidate sequences were subsequently submitted to NCBI’s Conserved Domain Database (CDD) to verify the presence of the PF00232 domain. Only sequences satisfying all filtering criteria were considered bona fide BrBGLU members and retained for downstream analyses. The completeness of the PF00232 domain was also assessed based on the CDD search results: full-length domain sequences were indicated by “—”, whereas incomplete domains at the C-terminus, N-terminus, or both termini were marked as “C”, “N”, and “NC”, respectively.

To investigate the evolutionary relationships among BrBGLU proteins, multiple sequence alignment was performed using MAFFT (v7.526). The resulting alignment was then used to construct a maximum-likelihood phylogenetic tree with IQ-TREE (version 3.1.2). Automated substitution model selection was conducted using the ModelFinder Plus (MFP) option, and branch support was assessed with 1000 ultrafast bootstrap replicates. All trees were visualized and annotated using the interactive online tool iTOL (https://itol.embl.de/, accessed on 20 April 2026).

### 4.2. Orthogroup Clustering, Pan-Genome Stratification, and GO Enrichment Analysis

All identified gene family members across the genome panel were subjected to orthogroup clustering using OrthoFinder v2.5.5 [59]. The resulting OGGs were subsequently stratified into pan-genome categories according to their occupancy across genomes, as summarized in the presence–absence matrix, which in turn was used to construct a heatmap using the TBtools to visualize the distribution of BGLUs across 21 *B. rapa* genomes [60]. OGGs were categorized as core (present in all 21 genomes), dispensable (present in 2–20 genomes), or private (unique to a single genome) based on their distribution across the accessions.

To characterize the functional distinctions between core and dispensable *BrBGLU* genes, GO enrichment analysis was carried out using ShinyGO (https://bioinformatics.sdstate.edu/go/, accessed on 20 April 2026), an interactive web-based tool for gene-set enrichment analysis [61]. Statistical significance was assessed using the hypergeometric test, and *p*-values were adjusted with the Benjamini–Hochberg method to control the false discovery rate (*FDR*). GO terms with an adjusted *FDR*-value < 0.05 were considered significantly enriched. The enrichment results were subsequently visualized and plotted using the clusterProfiler R package.

### 4.3. Analysis of Gene Duplication Modes

Gene duplication modes were systematically determined using the DupGen_finder pipeline [62]. The pipeline integrates BLASTP all-against-all alignment (*E*-value < 1 × e^−10^) with MCScanX-based synteny detection to identify duplicated gene pairs [63]. Based on their genomic distribution and syntenic relationships, duplicated genes were classified into five categories: whole-genome duplication (WGD), tandem duplication (TD, adjacent genes), proximal duplication (PD, separated by fewer than ten intervening genes), transposed duplication (TRD, involving ancestral and novel loci), and dispersed duplication (DSD). The outgroup species *Arabidopsis* was included as a reference to anchor syntenic blocks, enabling the distinction between ancestral polyploidization events and lineage-specific duplications.

### 4.4. RNA-Seq Data Processing and Expression Analysis

Publicly available RNA-seq datasets from *B. rapa* were obtained through NCBI BioProjects PRJNA529957, PRJNA185152, PRJNA641876, and PRJNA778186 [64,65,66,67]. These datasets comprised 54 samples collected from organs and tissues, including callus, stems, stem leaves, opened flowers, siliques, head leaves at different developmental stages, floral buds, pistils, embryos, and seed coats. Raw sequencing reads were trimmed using TrimGalore to remove low-quality bases and adapter sequences. The cleaned reads were then aligned to the *B. rapa* reference genome (Chiifu v4.0) employing HISAT2 [68]. Gene expression was quantified as TPM values. For the five genic male-sterility lines, RNA-seq data (BioProjects PRJDB9090, PRJNA310313, PRJNA376499, PRJNA473318, and PRJNA516468) were processed identically, and TPM values were derived from the same quantification pipeline [45,46,47,48,49]. Differentially expressed *BrBGLU* genes between fertile and sterile floral buds in the five male-sterile lines were identified using the DEGseq package in R [69]. Genes with a fold change ≥ 2 and an adjusted *p*-value ≤ 0.05 were considered significantly differentially expressed. The expression heatmaps were created using TBtools (version 2.056) [60]. Biological replicates varied among the male-sterile lines based on the available NCBI data. Three biological replicates were available for AB01 (PRJNA376499) and FT (PRJNA516468), whereas only one biological replicate was available for ms77B (PRJDB9090), AB (PRJNA310313), and Bcajh97-01A/B (PRJNA473318).

### 4.5. CRISPR/Cas9-Mediated Mutagenesis and Mutant Identification in B. rapa

Two sgRNAs targeting *BrBGLU10* (gRNA1: GTCGAGCTGGCTACGACATCGGG; gRNA2: CATGGGGACTTGTGTCACGATGG) were designed using the CRISPR-GE online tool (http://crispr.hzau.edu.cn/CRISPR2/, accessed on 22 April 2021) [70]. The sgRNA sequences were introduced into the binary vector pHSbdcas9i via Golden Gate assembly. The resulting construct was then delivered into *B. rapa* cv. GT-24 through *Agrobacterium tumefaciens*-mediated transformation [71,72]. Transgenic lines were identified by selection on medium containing hygromycin.

T_0_ transgenic plants were first acclimatized in soil and maintained in a growth chamber for 5–6 days, after which they were subjected to vernalization at 4 °C for three weeks and then returned to normal growth conditions. For each T_0_ plant, genomic DNA was isolated from cauline leaves using the EasyPure Universal Plant Genomic DNA Kit (TransGen Biotech, Beijing, China). PCR assays were carried out to confirm the presence of the transgenes encoding hygromycin resistance and *Cas9*. Following the onset of flowering, T0 plants were self-pollinated by bud pollination to harvest T_1_ seeds. The genomic regions encompassing the sgRNA target sites and predicted off-target sites were PCR-amplified from T_0_ and T_1_ genomic DNA, and the PCR products were gel-purified using the EasyPure Quick Gel Extraction Kit (TransGen Biotech). Purified fragments were then ligated into a cloning vector using the Lethal-Based Fast Cloning Kit (TransGen Biotech) according to the manufacturer’s instructions. All positive clones were subjected to Sanger sequencing to determine whether mutations had been introduced at the *BrBGLU10* target sites. For the sgRNA target sites, a minimum of ten independent cloned amplicons were sequenced per plant. For the potential off-target sites, six clones per sample were sequenced to assess the specificity of the editing. A complete list of the primers used in this study is provided in Table S5.

### 4.6. Pollen Viability and Silique Phenotyping

To assess male fertility, flowers from wild-type and T_1_ *BrBGLU10* mutant lines were fixed in Carnoy’s solution (ethanol: chloroform: acetic acid = 6:3:1, v/v/v) for 2 h. Pollen viability was subsequently evaluated using Alexander’s staining protocol [73]. Briefly, anthers were dissected and incubated in the staining solution containing malachite green, acid fuchsin, and orange G for 12 h. For each genotype, at least three independent plants were selected as biological replicates. From each plant, a minimum of five flowers were examined, with three technical replicates per flower. For silique measurements, ten siliques were harvested from the base to the top of the inflorescence for each plant, and at least three plants were included per genotype as biological replicates.

### 4.7. Statistical Analysis

Statistical analyses were performed using one-way analysis of variance (ANOVA) followed by Tukey’s multiple comparisons test for pairwise group comparisons, as indicated in the figure legends. Student’s *t*-test was used for comparisons between two groups where applicable. All statistical analyses were conducted using R software (version 4.4.1), and differences were considered statistically significant at *p* ≤ 0.05.

## 5. Conclusions

This pan-genomic analysis of the *BrBGLU* gene family across 21 *B. rapa* accessions identified 1840 members classified into five subgroups and 57 OGGs, with whole-genome duplication as the primary driver of gene family expansion. *BrBGLU10* and *BrBGLU56* were found to be specifically expressed in floral buds and differentially regulated between fertile and sterile lines. CRISPR/Cas9-mediated knockout of *BrBGLU10* resulted in substantial pollen abortion and severely reduced seed set, confirming its important role in pollen development and fertility. Collectively, these findings identify *BrBGLU10* as an important regulator of pollen development and a potential target for fertility-related applications via gene editing in *B. rapa* and related *Brassica* crops.

## Figures and Tables

**Table 1:** Overview of *BGLU* Gene Family Information of *B. rapa*.

Type	Sample	Name	*BGLU* No.	Protein Length			Molecular Weight (kDa)			Theoretical pI		
				Maximum	Minimum	Mean	Maximum	Minimum	Mean	Maximum	Minimum	Mean
Broccolieto	BRO	*B. rapa broccolieto*	95	998	121	513	114.52	13.75	58.39	9.15	4.95	6.82
Caixin	CXA	*B. rapa* ssp. *parachinensis*	75	1216	135	510	136.17	15.83	57.86	9.10	4.76	6.57
	CXB	*B. rapa* ssp. *parachinensis*	93	1358	87	506	155.48	10.13	57.65	9.37	4.49	6.79
Heading Chinese Cabbage	CCA	*B. rapa* ssp. *pekinensis*	93	989	107	512	113.46	12.32	58.35	9.09	4.91	6.80
	CCB	*B. rapa* ssp. *pekinensis*	82	988	100	509	113.34	12.27	58.09	9.16	5.00	6.79
	Chiifu	*B. rapa* ssp. *pekinensis*	83	1059	70	447	120.23	7.76	50.98	9.75	4.78	6.83
Mizuna	MIZ	*B. rapa* ssp. *nipposinica*	110	1240	128	472	140.85	14.79	53.80	9.46	4.93	6.84
Pak choi	AJ	*B. rapa* ssp. *chinensis*	82	1045	190	529	118.56	21.52	60.29	9.42	5.00	6.68
	HS	*B. rapa* ssp. *purpuraria*	86	1050	117	527	120.73	13.75	60.15	9.42	5.00	6.70
	PCA	*B. rapa* ssp. *chinensis*	91	1059	121	499	120.23	13.75	56.89	9.11	4.95	6.65
	PCB	*B. rapa* ssp. *chinensis*	86	1009	297	530	114.65	33.38	60.48	9.47	4.90	6.79
	TCA	*B. rapa* ssp. *chinensis*	90	1008	91	508	115.74	10.84	57.83	9.17	4.73	6.62
Sarsons	BraZ1	*B. rapa* ssp. *oleifera*	87	2642	70	535	306.12	7.76	60.99	9.44	4.80	6.84
	OIA	*B. rapa* ssp. *oleifera*	91	1059	70	494	120.23	7.81	56.33	9.49	4.38	6.70
	OIB	*B. rapa* ssp. *oleifera*	75	1462	89	463	162.23	9.66	52.63	9.23	4.85	6.79
	OIC	*B. rapa* ssp. *oleifera*	90	1335	70	496	148.74	7.76	56.54	9.16	4.91	6.70
Turnip	ECD04	*B. rapa* subsp. *rapifera*	78	1033	59	474	115.05	7.41	53.95	9.99	4.64	6.55
	TUA	*B. rapa* ssp. *rapa*	70	984	86	364	111.38	9.57	41.42	9.38	4.78	6.51
	TUE	*B. rapa* ssp. *rapa*	100	1361	121	503	151.75	13.75	57.26	9.14	4.91	6.83
Wutacai	WTC	*B. rapa* ssp. *narinosa*	82	1059	111	419	120.23	12.52	47.67	9.40	4.81	6.75
Unknown	TBA	*unknown*	101	850	121	490	97.12	13.75	55.87	9.19	4.91	6.85

## Data Availability

The datasets generated during and/or analyzed during the current study are available from the corresponding author on reasonable request.

## Supplementary Materials

The following supporting information can be downloaded at https://www.mdpi.com/article/10.3390/plants15172579/s1, Figure S1: Heatmap of differentially expressed *BrBGLUs* across five genic male sterility (GMS) lines. The heatmap illustrates the transcript abundance of *BrBGLUs* in male sterile lines ms77B, FT, AB, AB01, and Bcajh97-01A/B. For ms77B, four progressive floral bud stages—pre-tetrad, tetrad, post-tetrad, and mature pollen—were examined for both fertile (F1–F4) and sterile (S1–S4) plants. For lines FT, AB, and AB01, fertile (“F”) and sterile (“S”) floral buds are compared. For line Bcajh97-01A/B, five pollen developmental stages are represented: before tetrad (FS1/SS1), tetrad (FS2/SS2), uninucleate (FS3/SS3), binucleate (FS4/SS4), and mature pollen (FS5/SS5), with “F” and “S” denoting fertile and sterile conditions, respectively. For RNA-seq data, three biological replicates were available for AB01 and FT, whereas only one biological replicate was available for ms77B, AB, and Bcajh97-01A/B; Table S1: Amino acid sequences of *BrBGLU* family members; Table S2: Orthogroup analysis of *BrBGLU* genes; Table S3: Detailed information on duplication types and pangenomic classification of *BGLU* genes in *B. rapa*; Table S4: Expression levels of *BrBGLUs* across five genic male sterility (GMS) lines. The heatmap illustrates the transcript abundance of *BrBGLUs* in male sterile lines ms77B, FT, AB, AB01, and Bcajh97-01A/B. For ms77B, four progressive floral bud stages—pre-tetrad, tetrad, post-tetrad, and mature pollen—were examined for both fertile (F1–F4) and sterile (S1–S4) plants. For lines FT, AB, and AB01, fertile (“F”) and sterile (“S”) floral buds are compared. For line Bcajh97-01A/B, five pollen developmental stages are represented: before tetrad (FS1/SS1), tetrad (FS2/SS2), uninucleate (FS3/SS3), binucleate (FS4/SS4), and mature pollen (FS5/SS5), with “F” and “S” denoting fertile and sterile conditions, respectively. PCC indicates Pearson correlation coefficient. Red font indicates differentially expressed genes in at least one male-sterile line. Red background indicates up-regulated genes. Cyan background indicates down-regulated genes; Table S5: List of primers used in this study.

## Abbreviations

The following abbreviations are used in this manuscript:

GH1	Glycoside hydrolase family 1
BGLUs	Glycoside hydrolase family
BrBGLUs	*Brassica rapa* β-glycosidase genes
ABA	Abscisic acid
GO	Gene Ontology
HMM	Hidden Markov Model
sgRNA	Single-guide RNA
PAM	Protospacer adjacent motif
TPM	Transcripts Per Million
OGGs	Orthologous gene groups
